# Potential impact of the COVID-19 pandemic on HIV, tuberculosis, and malaria in low-income and middle-income countries: a modelling study

**DOI:** 10.1016/S2214-109X(20)30288-6

**Published:** 2020-07-13

**Authors:** Alexandra B Hogan, Britta L Jewell, Ellie Sherrard-Smith, Juan F Vesga, Oliver J Watson, Charles Whittaker, Arran Hamlet, Jennifer A Smith, Peter Winskill, Robert Verity, Marc Baguelin, John A Lees, Lilith K Whittles, Kylie E C Ainslie, Samir Bhatt, Adhiratha Boonyasiri, Nicholas F Brazeau, Lorenzo Cattarino, Laura V Cooper, Helen Coupland, Gina Cuomo-Dannenburg, Amy Dighe, Bimandra A Djaafara, Christl A Donnelly, Jeff W Eaton, Sabine L van Elsland, Richard G FitzJohn, Han Fu, Katy A M Gaythorpe, William Green, David J Haw, Sarah Hayes, Wes Hinsley, Natsuko Imai, Daniel J Laydon, Tara D Mangal, Thomas A Mellan, Swapnil Mishra, Gemma Nedjati-Gilani, Kris V Parag, Hayley A Thompson, H Juliette T Unwin, Michaela A C Vollmer, Caroline E Walters, Haowei Wang, Yuanrong Wang, Xiaoyue Xi, Neil M Ferguson, Lucy C Okell, Thomas S Churcher, Nimalan Arinaminpathy, Azra C Ghani, Patrick G T Walker, Timothy B Hallett

**Affiliations:** a Medical Research Council Centre for Global Infectious Disease Analysis, Abdul Latif Jameel Institute for Disease and Emergency Analytics, Imperial College London, London, UK; b Department of Statistics, University of Oxford, Oxford, UK

## Abstract

**Background:**

COVID-19 has the potential to cause substantial disruptions to health services, due to cases overburdening the health system or response measures limiting usual programmatic activities. We aimed to quantify the extent to which disruptions to services for HIV, tuberculosis, and malaria in low-income and middle-income countries with high burdens of these diseases could lead to additional loss of life over the next 5 years.

**Methods:**

Assuming a basic reproduction number of 3·0, we constructed four scenarios for possible responses to the COVID-19 pandemic: no action, mitigation for 6 months, suppression for 2 months, or suppression for 1 year. We used established transmission models of HIV, tuberculosis, and malaria to estimate the additional impact on health that could be caused in selected settings, either due to COVID-19 interventions limiting activities, or due to the high demand on the health system due to the COVID-19 pandemic.

**Findings:**

In high-burden settings, deaths due to HIV, tuberculosis, and malaria over 5 years could increase by up to 10%, 20%, and 36%, respectively, compared with if there was no COVID-19 pandemic. The greatest impact on HIV was estimated to be from interruption to antiretroviral therapy, which could occur during a period of high health system demand. For tuberculosis, the greatest impact would be from reductions in timely diagnosis and treatment of new cases, which could result from any prolonged period of COVID-19 suppression interventions. The greatest impact on malaria burden could be as a result of interruption of planned net campaigns. These disruptions could lead to a loss of life-years over 5 years that is of the same order of magnitude as the direct impact from COVID-19 in places with a high burden of malaria and large HIV and tuberculosis epidemics.

**Interpretation:**

Maintaining the most critical prevention activities and health-care services for HIV, tuberculosis, and malaria could substantially reduce the overall impact of the COVID-19 pandemic.

**Funding:**

Bill & Melinda Gates Foundation, Wellcome Trust, UK Department for International Development, and Medical Research Council.

## Introduction

The COVID-19 pandemic, and actions taken in response to it, will have far-reaching consequences on other diseases, poverty, food security, and economic growth.^1^ In low-income and middle-income countries, a particular concern is the potential impact on three major health priorities, specifically, HIV, tuberculosis, and malaria, due to a possible disruption to health services. Many low-income and middle-income countries have high burdens of these three diseases, and millions of people depend on large-scale programmes to control and treat them.^2^, ^3^, ^4^ In recent years, substantial progress has been made in reducing the burden of HIV, tuberculosis, and malaria, and ambitious targets have been set for reaching very low levels of burden by 2030, as part of the Sustainable Development Goals.^5^ Interruptions to control programmes could result in major setbacks, compounding the direct impact of COVID-19.

We conceptualise the potential impact on HIV, tuberculosis, and malaria programmes as arising predominantly from disruptions to the usual activities and services due to COVID-19. These disruptions include mitigation strategies being undertaken in response to the COVID-19 pandemic, leading to the scaling back of certain activities and care-seeking; reduced capabilities of the health system due to overwhelmingly high demand for the care of patients with COVID-19; and interruptions to the supply of commodities as a result of effects on both domestic and international supply chains. We aimed to estimate the extent to which such disruptions in low-income and middle-income countries with high burdens of HIV, tuberculosis, and malaria could lead to additional loss of life over the next 5 years.

**Research in context**

**Evidence before this study**

The COVID-19 pandemic could have major adverse effects on the provision of health services for other major infectious diseases in low-income and middle-income countries. We searched PubMed for articles published up to May 14, 2020, with the terms ((“COVID-19”[Title] OR “SARS-CoV-2”[Title] OR “coronavirus”[Title]) AND (“HIV”[All Fields] OR “tuberculosis”[All Fields] OR “TB”[All Fields] OR “malaria”[All Fields]) AND (“Data”[Title] OR “Model”[Title])). No language restrictions were applied. Our search found no results for peer-reviewed studies providing quantitative analyses of such effects for HIV, tuberculosis, or malaria.

**Added value of this study**

We provide the first study of the potential combined impact of disruptions caused by COVID-19 on HIV, tuberculosis, and malaria in low-income and middle-income countries. The estimates are designed to be broadly comparable between diseases, readily extrapolated to other countries, and directly compared with the potential impact of the COVID-19 pandemic itself.

**Implications of all the available evidence**

Our analysis indicates that the COVID-19 pandemic could cause a substantial increase in HIV, tuberculosis, and malaria deaths in settings with high burdens of those diseases. The impact varies according to the extent to which interventions against COVID-19 cause prolonged disruptions to activities, and how successfully those measures suppress transmission of severe acute respiratory syndrome coronavirus 2, and avoid the health system being overwhelmed. The impact on HIV, tuberculosis, or malaria could be minimised by maintaining core services: continued access to antiretrovirals, maintenance of tuberculosis diagnosis and treatment, and early resumption of the distribution of long-lasting insecticide-treated nets.

## Methods

### Study design and procedures

Based on a study by Walker and colleagues,^1^ we assumed a basic reproduction number (*R*_0_; the number of new infections caused by a single infection in a wholly susceptible population) of 3·0 for severe acute respiratory syndrome coronavirus 2 (SARS-CoV-2), the causative agent of COVID-19. We then constructed four scenarios that describe a range of possible trajectories for the resulting COVID-19 pandemic in low-income and middle-income countries (two representative settings for each disease) with respect to the effect that interventions have on reducing the effective reproduction number (*R*_t_; the number of new infections caused by a single infection at time *t* during an epidemic; table 1). The specification of the mitigation scenario was chosen to approximate the maximum reduction in the final size of the pandemic that can be achieved with partially effective and non-permanent interventions.^1^ The timings of the pandemic are representative of settings in which there was a total of 0·1 deaths per million population due to COVID-19 by April 12, 2020, which was the case for most countries in sub-Saharan Africa. In this context, deaths from COVID-19 in the no action scenario would peak in July, 2020.

**Table 1:** COVID-19 scenarios

	Assumptions*
No action	No substantial interventions in response to the COVID-19 pandemic
Mitigation	Interventions capable of reducing the COVID-19 *R*_t_ by 45% are used for 6 months (eg, physical distancing rules, no mass public gatherings, and home-working when possible)
Suppression–lift	Interventions capable of reducing the COVID-19 *R*_t_ by 75% are implemented for 2 months, then lifted (eg, a full lockdown intervention, including closure of non-essential businesses and schools, and no non-essential travel or time spent outside of the home)
Suppression	Interventions capable of reducing *R*_t_ by 75% are implemented for 1 year (implicitly assuming that pharmacological interventions become available by that time and that therefore there is no large COVID-19 pandemic in the next 5 years); this scenario is subdivided into well managed suppression and unmanaged suppression, which are distinguished by their effects on HIV, tuberculosis, and malaria

*R*_t_=effective reproduction number.

* For all scenarios, we also assume that the COVID-19 *R*_t_ is reduced by 20% irrespective of any intervention due to a spontaneous reduction in social contacts.

The course of each possible trajectory was divided into periods during which different types of disruptions to services might occur, either due to COVID-19 interventions limiting activities, or due to the high demand on the health system due to the COVID-19 pandemic. We assumed that there was high demand on the health system when the number of people requiring non-critical care in hospitals for COVID-19 exceeded 50% of the prevailing capacity of hospitals, and that there was extremely high demand when that number exceeded 100% of capacity.

We made assumptions about how the programmes for HIV, tuberculosis, and malaria prevention and treatment would be affected (table 2). These assumptions were made in two separate categories: factors related to COVID-19 interventions limiting other activities and factors related to demand on the health system. Multiple types of disruption could occur simultaneously in the simulations and the combination and extent of each type of disruption varied between the different COVID-19 scenarios (table 1). We also created two versions of the suppression scenario that had the same COVID-19 outcomes but different effects on other health services (table 2). In one scenario, well managed suppression, we assumed that over this long period of suppression, no more disruption was caused than during the short-term application of mitigation or suppression interventions. In the unmanaged suppression scenario, we assumed that substantial obstacles to the provision of other health services accumulated during this long period of substantial intervention.

**Table 2:** Assumptions of how HIV, tuberculosis, and malaria programmes will be affected by COVID-19 in different response scenarios

	**Limitations on activities**		**Demand on health system**		**During period of recovery**
	Mitigation or well managed suppression	Unmanaged suppression*	High demand	Extremely high demand†	
HIV	Care seeking reduced: rate of new ART initiations reduced by 25%, 2% of individuals on ART become virally unsuppressed per month; prevention services partially suspended: no new VMMC, no new PrEP enrolments; reduced social contact: 10% reduction in chance of acquiring new sexual partner	Care seeking reduced: rate of new ART initiations reduced by 50%, 1% of individuals on ART stop per month due to inability to attend appointments; prevention services suspended: no renewals of PrEP prescriptions	Care, medicine, and diagnosis less available at facilities: no new ART initiations, 25% of individuals on ART pre-pandemic have their ART use interrupted, an additional 10% of individuals on ART become virally unsuppressed due to lack of viral load testing; prevention services suspended: no new VMMC or PrEP enrolments; no renewals of PrEP prescriptions	Supply of drugs and commodities interrupted: 50% of individuals on ART pre-pandemic have their ART use interrupted, condom use reduced by 50%	All services and behaviours resume to pre-pandemic levels immediately
Tuberculosis	Care seeking reduced: diagnosis rates decrease by 25% compared with pre-pandemic levels, patient delays before the first presentation to care increased by 25% compared with pre-pandemic levels; displacement of diagnostic resources: diagnosis rate for tuberculosis and drug-resistant tuberculosis decreased by a further 45% due to non-availability of Xpert MTB/Rif diagnostics, yielding overall reduction of 70%; reduced social contact: transmission reduced by 10%	Care seeking reduced: tuberculosis and drug-resistant tuberculosis diagnosis rates decrease by 50%, patient delays before first presentation to care increased by 50% compared with pre-pandemic levels; displacement of diagnostic resources: diagnosis rate for tuberculosis and drug-resistant tuberculosis decreased by a further 20% due to non-availability of Xpert MTB/Rif diagnostics, yielding overall reduction of 70%; prevention services suspended: no new IPT for people with HIV	Care, medicine, and diagnosis less available at facilities: treatment completion rates decrease by 25% for first-line and second-line treatment compared with pre-pandemic levels, patient delays before first presentation to care increased by 50% compared with pre-pandemic levels, drug-resistant tuberculosis diagnosis rates decrease by 50% compared with pre-pandemic levels; prevention services suspended: no new IPT for people with HIV	Supply of drugs interrupted: treatment initiation rates decrease to 50% of pre-pandemic levels; drug-resistant tuberculosis diagnosis rates decrease to 0% (also impacted by diagnosis not being available)	All services and behaviours resume to pre-pandemic levels immediately
Malaria	Care seeking reduced: treatment of clinical cases reduced by 25% compared with pre-pandemic levels; prevention services partially suspended: LLIN mass distribution continues as normal, SMC at 50% of normal coverage	Care seeking reduced: treatment of clinical cases reduced by 50% compared with pre-pandemic levels; prevention services suspended: LLIN mass campaigns halted, SMC halted	Care, medicine, and diagnosis less available at facilities: treatment of clinical cases reduced by 25% compared with pre-pandemic levels; prevention services suspended: LLIN mass campaigns halted, SMC halted	Supply of drugs interrupted: treatment of clinical cases reduced by 50% compared with pre-pandemic levels	Treatment of clinical cases remains at reduced level for 2 months; all other services and behaviours resume to pre-pandemic levels immediately

ART=antiretroviral therapy. VMMC=voluntary medical male circumcision. PrEP=pre-exposure prophylaxis. IPT=isoniazid preventive therapy. LLIN=long-lasting insecticide-treated nets. SMC=seasonal malaria chemoprevention.

* These changes are assumed to be in addition to those that occur during the mitigation interventions.

† These changes are assumed to be in addition to those that occur during the period of high demand.

These assumptions were designed to give a consistent representation of the extent of disruption in the programme elements for each disease, to allow the results to be comparable. Further specific justifications for each change are provided in the appendix (pp 7–9). Routine services for prevention (voluntary medical male circumcision, pre-exposure prophylaxis, long-lasting insecticide-treated nets [LLINs], and seasonal malaria chemoprevention) were assumed to be at least partially suspended during any disruption; provision of ongoing treatment (for HIV or tuberculosis) or new acute treatment (for malaria) was reduced by 25% and 50% in the high and extremely high periods of health system demand, respectively; and treatment for individuals who were newly seeking care (HIV or tuberculosis testing and treatment) was reduced by 25% in the mitigation and well managed suppression scenarios, and by 50% during the unmanaged suppression scenario. Diagnosis and treatment rates for tuberculosis were reduced further than for the other conditions, because of the additional impact of Xpert MTB/Rif, a molecular diagnostic tool for tuberculosis, potentially being repurposed for COVID-19 diagnosis. For HIV and tuberculosis, we also assumed that the social distancing measures introduced through the COVID-19 response would result in 10% fewer sexual and close contacts for transmission of the respective diseases.

The health impact of these disruptions was estimated using a separate model for each disease (appendix pp 7–9).^6^, ^7^, ^8^ Each model was applied to two contrasting disease burden settings chosen to capture a range of impact severity due to health system disruption. For HIV, the first setting was a very high HIV prevalence setting (20% among 15–49-year-olds in 2018) typical in southern Africa; the second was a high HIV prevalence setting (9% among 15–49-year-old adults in 2018) typical in eastern Africa. For tuberculosis, the first setting was a very high burden setting (tuberculosis incidence of 520 per 100 000 population in 2018) typical in southern Africa; the second was a moderate burden setting (tuberculosis incidence of 45 per 100 000 population in 2018) typical in South America. For malaria, the first setting was a generic high malaria burden setting with seasonality of transmission typical of a west African country (around 386 000 malaria cases per million people in 2018); the second was a generic moderate burden setting with seasonality of transmission typical of a country in eastern Africa (around 7000 malaria cases per million people in 2018); both settings were assumed to have scheduled LLIN campaigns in 2020.

Deaths from HIV and tuberculosis are reported separately even though these can overlap in reality. The total excess deaths caused by either HIV or tuberculosis in these models is estimated to be equal to approximately 65% of the sum of each cause when computed separately.

### Outcomes

The impact of the COVID-19 pandemic and associated disruptions was quantified as the additional deaths and the additional years of life that are lost, compared with a scenario in which there was no COVID-19 pandemic or associated disruptions (and programmes continued or expanded in the manner that would have been expected in this period otherwise), over a period of 1 year and a period of 5 years. Outcomes pertain to the whole population (all ages and sexes). The years of life lost if a person dies due to COVID-19, or due to HIV, tuberculosis, or malaria, were computed with respect to the life expectancy for that age of person in the respective country setting. When comparing scenarios, we defined the worst scenario as that for which the greatest number of additional deaths were projected over 5 years.

### Role of the funding source

The funders of the study had no role in study design, data collection, data analysis, data interpretation, or writing of the report. The corresponding author had full access to all the data in the study and had final responsibility for the decision to submit for publication.

## Results

**Figure 1:**
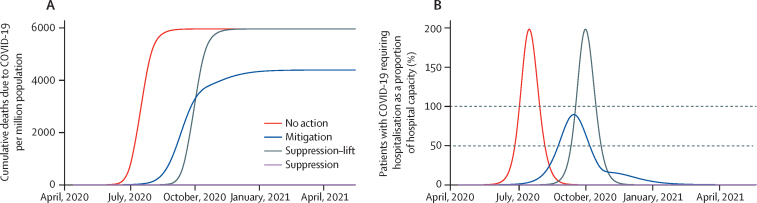
Deaths due to COVID-19 and hospital capacity in each pandemic scenario (A) Cumulative deaths due to COVID-19 per million population. (B) Patients with COVID-19 requiring non-critical care in hospital as a proportion of total hospital capacity. Dashed lines indicate the thresholds of high (50%) and extremely high (100%) health system demand.

The projections for cumulative deaths due to COVID-19 over the course of the pandemic are shown in figure 1. In the counterfactual no action scenario, the direct deaths due to COVID-19 were predicted to occur mostly between June and August (although these dates are sensitive to when the pandemic begins) with an estimated 6000 deaths per million population. In this scenario, 30% of projected COVID-19 deaths would be due to lack of supportive care due to hospital capacity being exceeded (ie, 30% fewer deaths would occur if hospital capacity was not limited). In the mitigation scenario, we projected a lower number of COVID-19 deaths (around 4400 per million population), because the pandemic curve would be flatter, with a lower peak and lasting for longer than the no action scenario. The flatter curve occurs because a small amount of the population become infected and immune during the mitigation period, so that when restrictions are removed, the peak of the epidemic is curbed. As such, the number of patients needing care at any one time does not exceed hospital capacity. The relative number of deaths in the no action and mitigation scenarios depends on the hospital capacity assumed and the extent to which the mitigation interventions result in more people receiving treatment; if hospital capacity is very low, for instance, then we would project a smaller difference between the mitigation intervention and no action scenarios.

The aforementioned projections are sensitive to the value of *R*_0_ that is assumed for SARS-CoV-2 (appendix p 3). When the pandemic spreads in a population with no immunity (ie, in the no action or suppression–lift scenarios), a lower *R*_0_ value (2·5) leads to a smaller pandemic and a lower total number of deaths, whereas a higher value (3·5) leads to a greater total number of deaths. For scenarios in which interventions are insufficient to lower *R*_t_ to less than 1 (ie, the mitigation scenario), the total number of COVID-19 deaths will be sensitive to both *R*_0_ and the extent to which a degree of immunity is established in the population by the time that the measures are lifted. As a result, our mitigation scenario (which represents a 45% reduction in contact rates for a 6-month period) resulted in a higher number of deaths for both a lower and a higher assumed value for *R*_0_. With an *R*_0_ of 3·5, the epidemic had a higher *R*_t_ throughout the period of mitigation and therefore spread continued. By contrast, with an *R*_0_ of 2·5, the epidemic was better controlled during the period of mitigation but then had a large peak in cases that overwhelmed health care capacity when mitigation was lifted, driven by the low levels of immunity acquired within the population at this point.

In the suppression scenario, we assumed that the interventions put in place reduced the *R*_t_ of COVID-19 to less than 1 and therefore the pandemic was controlled. In this scenario, we assumed that those interventions were maintained until other means of controlling the pandemic, such as pharmaceutical interventions, became available. In the suppression–lift scenario, we projected a delayed pandemic of a size similar to that in the no action scenario, because we assumed the risk of transmission would be the same after the lifting of interventions. However, if additional interventions or strategies are found to maintain control of the virus then this assumption would not hold and the suppression–lift scenario would be more similar to the suppression scenario.

**Figure 2:**
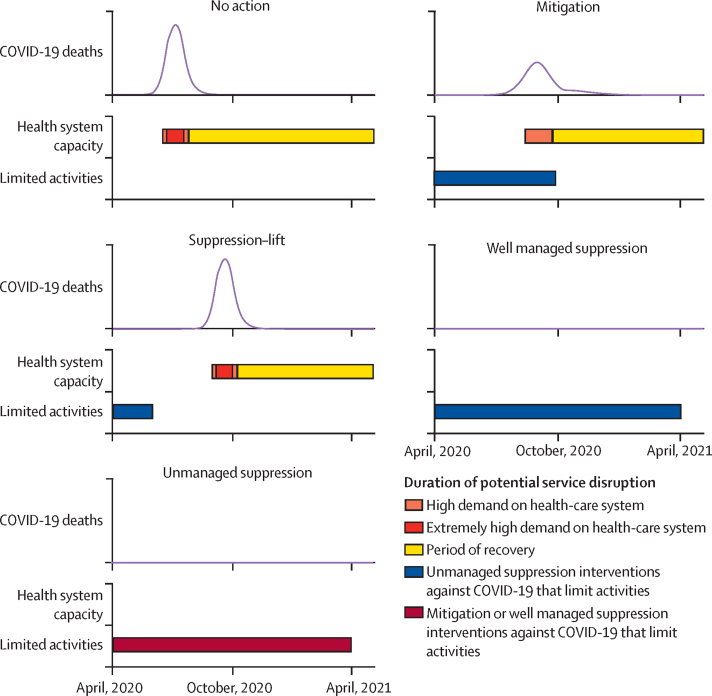
Patterns of disruption to health care in each pandemic scenario Black lines show the number of COVID-19 deaths per day for each. The periods indicated with the shaded bars show the timings of the different types of disruption.

Under our modelling assumptions, these pandemic scenarios induce different patterns of disruption (figure 2). In the no action scenario, there is a period of 6 weeks during which the health system is under high demand, within which extremely high demand is experienced for 4 weeks. In the mitigation scenario, there is a 6-month period of disruption caused by the interventions, in which the last 6 weeks are a period of high demand on the health system, but there is no period of extremely high demand. In the suppression–lift scenario, there is a 2-month period of disruption caused by the suppression intervention and the same sequence of health system demand as in the no action scenario, but this is delayed by 11 weeks. In the two scenarios for suppression interventions, there is no period of high demand on the health system (because there is no large pandemic) but there is a 12-month period of disruption caused by interventions, which could be either well managed or unmanaged.

The patterns of disruption are also sensitive to the assumed value of *R*_0_ for SARS-CoV-2. In the no action or suppression–lift scenarios, if the *R*_0_ of SARS-CoV-2 were lower (2·5), then the extent of the disruptions would last longer; if the *R*_0_ were higher (3·5), the disruptions could be shorter (though potentially of a more intense nature; appendix p 3). These differences will depend on the definitions of high demand on the health system, but for the definitions used here, the differences are small—eg, the difference in the number days of the health system being in a state of high or extremely high demand in the no action scenario with an *R*_0_ of 2·5 or 3·5 is 5 days. The patterns of disruptions are more uncertain in scenarios in which there is a mitigation intervention that attempts to move *R*_t_ towards 1·0 (appendix p 4). In the mitigation scenario, an *R*_0_ of 3 leads to a lower peak in cases and therefore a lower peak in deaths, whereas different values of *R*_0_ or different extents of mitigation would lead to different results: lower or higher values of *R*_0_ in scenarios that had the same mitigation intervention would experience a period of extremely high health system demand, and weaker and stronger mitigation interventions used in an epidemic with an *R*_0_ of 3 would also lead to a period of extremely high health system demand. In each case, this is because there is a finely balanced trade-off between the extent to which a mitigation intervention leads to a slow build-up of immunity that avoids a so-called second wave, and the extent to which it does not impede transmission sufficiently to curb a first wave (appendix p 4).

**Figure 3:**
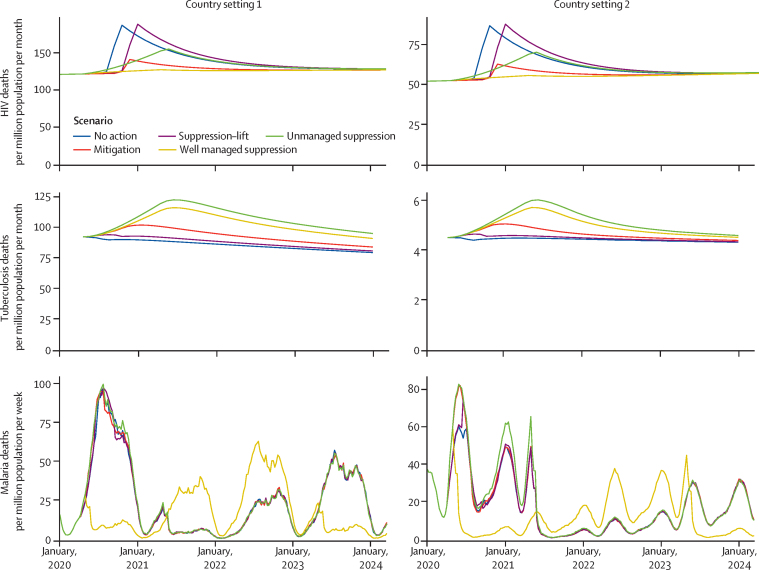
Additional deaths due to HIV, tuberculosis, and malaria resulting from the impact of COVID-19 For HIV, setting 1 is a very high HIV prevalence setting (20% among 15–49-year-olds in 2018) typical in southern Africa; setting 2 is a high HIV prevalence setting (9% among 15–49-year-old adults in 2018) typical in eastern Africa. For tuberculosis, setting 1 is a very high burden setting (tuberculosis incidence of 520 per 100 000 population in 2018) typical in southern Africa; setting 2 is a moderate burden setting (tuberculosis incidence of 45 per 100 000 population in 2018) typical in South America. For malaria, setting 1 is a generic high malaria burden setting with seasonality of transmission typical of a west African country (around 386 000 malaria cases per million people in 2018); setting 2 is a generic moderate burden setting with seasonality of transmission typical of a country in eastern Africa (around 7000 malaria cases per million people in 2018).

Based on the projected disruptions due to COVID-19, we modelled the number of additional deaths due to HIV, tuberculosis, and malaria (figure 3, appendix p 4). The greatest increase in HIV deaths was predicted to be caused by forced interruptions to antiretroviral therapy (ART) for some individuals, which was assumed to occur during the periods of extremely high health system demand (in the no action and suppression–lift scenarios). Smaller modelled impacts are due to a reduction in new ART initiations (in the mitigation scenario) and a gradual accumulation of individuals not taking ART (in the unmanaged suppression scenario; appendix p 4). HIV deaths are predicted to remain elevated after the period of disruption because the reduction in CD4 cell count for some individuals is assumed to lead to an increased risk of AIDS for some time after ART is reinitiated. In the worst scenario (ie, no action or suppression–lift in country setting 1, for which the greatest number of additional deaths are projected over 5 years), 10% more deaths due to HIV were projected to occur over 5 years than would occur without the disruptions.

The greatest increase in deaths due to tuberculosis is predicted to be due to the prolonged periods of reduced diagnosis and treatment of new tuberculosis cases that are assumed to occur in the suppression (both well managed and unmanaged) scenarios. The period of extremely high demand on the health system is predicted to have a small effect on increasing tuberculosis deaths because it is short and the effects are overcome during the recovery phase. The disruption is predicted to lead to an increase in tuberculosis deaths for several years because the disruptions leave individuals untreated for longer, leading to more transmission and more cases in later years. In the worst scenario (suppression in country setting 1), 20% more deaths due to tuberculosis were projected to occur over 5 years than would occur without the disruptions.

The greatest increase in deaths due to malaria deaths was predicted to be due to LLINs not being distributed before a peak in malaria transmission (which occurs in all scenarios except well managed suppression). The projected deaths due to malaria could be lower in the following years than they would have been without disruption because it is assumed that the LLINs are distributed in the recovery phase and therefore more people benefit from newer LLINs in the later years (simply because in 2021, the nets would be 1 year old if they had been delivered in 2020 as planned; figure 3). We project a greater number of additional deaths over 5 years caused by the disruptions in country setting 2 compared with country setting 1, despite a lower overall malaria burden in country setting 2, because the timing of the disruptions has a larger degree of overlap with the malaria transmission season in that region, and it also coincides with the planned schedule of net distribution in country setting 2. In the worst scenario (all except well managed suppression in country setting 2), we predict 36% more deaths over 5 years than would have occurred without the COVID-19 disruptions.

**Figure 4:**
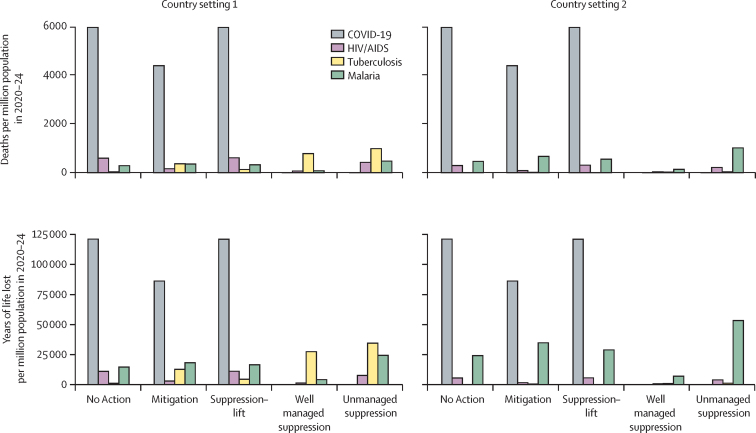
Additional deaths (upper panels) and years of life lost (lower panels) due to the COVID-19 pandemic and related disruption to care for HIV, tuberculosis, and malaria in 2020–24 For HIV, setting 1 is a very high HIV prevalence setting (20% among 15–49-year-olds in 2018) typical in southern Africa; setting 2 is a high HIV prevalence setting (9% among 15–49-year-old adults in 2018) typical in eastern Africa. For tuberculosis, setting 1 is a very high burden setting (tuberculosis incidence of 520 per 100 000 population in 2018) typical in southern Africa; setting 2 is a moderate burden setting (tuberculosis incidence of 45 per 100 000 population in 2018) typical in South America. For malaria, setting 1 is a generic high malaria burden setting with seasonality of transmission typical of a west African country (around 386 000 malaria cases per million people in 2018); setting 2 is a generic moderate burden setting with seasonality of transmission typical of a country in eastern Africa (around 7000 malaria cases per million people in 2018).

Overall, the predicted magnitude of the impact on HIV, tuberculosis, and malaria was broadly similar, in terms of additional deaths and years of life lost (figure 4; appendix pp 1–2). We expect a substantial overlap in the deaths counted under HIV and tuberculosis in country setting 1, as noted in the Methods.

The predicted number of additional deaths and years of life lost due to HIV, tuberculosis, and malaria over 5 years is smaller but of a similar magnitude to those that would be expected to be caused by the COVID-19 pandemic itself (figure 4). Settings in which malaria prevention interventions are particularly heavily affected could see an increase in the years of life lost due to malaria to approximately 40% of the years of life lost due to COVID-19 in the mitigation scenario. In any setting that has as high a burden of HIV, tuberculosis, and malaria as in each of our case study countries, the number of years of life lost due to the indirect effects of COVID-19 on these three diseases could be up to 60% of the years of life lost due to COVID-19 directly. In lower burden settings, the magnitude of the impact of disruptions is accordingly predicted to be much less than the impact of the COVID-19 pandemic itself.

## Discussion

In settings with high burdens of HIV, tuberculosis, or malaria, disruptions during the COVID-19 pandemic could cause an increase in deaths due to HIV of up to 10%, due to tuberculosis of up to 20%, and due to malaria of up to 36%, over 5 years compared with if no COVID-19 pandemic occurred. In regions with a high burden of all three diseases, disruptions could cause an additional number of years of life lost over 5 years that is less than but of the same order of magnitude as the direct impact from COVID-19. Therefore, in settings with high burdens of HIV, tuberculosis, or malaria, maintaining a continuity of services and recovering programmes should be a high priority to reduce the broader health impact of the COVID-19 pandemic.

This indirect impact of the pandemic might be largely avoided through maintenance of core programme elements and recovery campaigns. For HIV, individuals receiving ART should continue to access treatment even in periods of highest health system demand (eg, via multimonth prescriptions or dispensing away from health facilities).^9^ For tuberculosis, routes for individuals to seek care and diagnosis must be provided despite interventions that promote social distancing. For malaria, preventative measures must be prioritised, ensuring LLINs and prophylactic treatments, such as mass drug distribution or seasonal malaria chemoprevention, are conducted at scale as soon as possible.

Our results underscore the extraordinarily difficult decisions facing policy makers. Well managed, long-term suppression interventions could avert the most deaths through avoiding a COVID-19 pandemic; however, if the interventions are not well managed, they could lead to a large spike in deaths from other causes. In either case, suppression interventions will have enormous impacts of other types, in the worst cases risking jobs, livelihoods, food security, and more. If such suppression interventions are not feasible, then mitigation-type interventions might lead to fewer overall deaths (including deaths due to COVID-19 and other diseases) than in other scenarios. However, a less effective or less well managed intervention could still result in a high number of COVID-19 deaths and could lead to a greater increase in deaths from other causes. An intense but short period of suppression intervention (the suppression–lift scenario) could generate a valuable delay in the pandemic that provides the opportunity to increase hospital capacity and engineer reductions in contacts. Yet, if such changes were not possible, then the impact of the pandemic would simply be compounded by the disruptions incurred during the initial period of intervention. Furthermore, it is not known whether the risk of COVID-19 deaths that could be directly attributed to the continuation of some services, such as LLIN distribution, would exceed the benefit that might be gained in reduced deaths from other causes.

The major uncertainties in this analysis can be classified into three groups: uncertainty about the scale of the COVID-19 pandemic; uncertainty about the extent to which other disease programmes will actually be disrupted; and uncertainty about how those disruptions will impact on population health. It should be noted that producing a reliable modelling analysis at a time when data are still scarce is difficult. In particular, our understanding is rapidly evolving with regard to the risk of mortality upon infection with SARS-CoV-2, how this is affected by underlying comorbidities (including for those co-infected with HIV, tuberculosis, or malaria), age, or setting (ie, how mortality might be different in Africa to that in China, Europe, and the USA), and the possible effects of treatment.^10^ We do not have precise knowledge of the transmissibility of SARS-CoV-2 (represented by the *R*_0_ value) or a detailed understanding of how this varies across settings. Meanwhile, the extent to which policies that are implemented are successful in reducing transmission is also uncertain and likely to vary by context. Our analyses show that uncertainty in these two factors can be conceptualised as a continuum between a no-action epidemic and a suppressed epidemic: a higher *R*_0_ value or a lower degree of mitigation moves epidemics closer to a no-action type, which induces a high strain on the health system over a shorter period; a lower *R*_0_ value or a higher degree of mitigation is more likely to resemble a suppressed-type epidemic, which will maintain burden at lower levels while measures are maintained but results in a second wave of infections if those interventions are removed. As a result, the scenarios that have been constructed do not cover all possible eventualities, but they do illustrate the trade-offs between the extent of disruption due to interventions and periods of high health system demand.

Although the actual effects on disease programmes remain unclear, some community-based programmes are already being scaled back (appendix pp 7–9)^11^ and experience in high-income settings has shown a substantial reduction in engagement with regular medical care during recent periods of high health system demand.^12^ Another factor that could diminish capability during the periods of highest demand is health-care staff shortages due to COVID-19 illness.^13^ Disruptions to supply chains have not yet occurred on a large scale, although it is a credible threat given the reliance on international trade routes that could be affected by economic factors and travel restrictions. Of note, this type of effect has been observed before—eg, during the Ebola epidemic in Guinea in 2014, more additional people died from malaria that year due to fewer malaria treatments being administered than died from Ebola.^14^

Estimating the impact of some types of disruption on population health, especially over longer time periods, is restricted by the paucity of data on relevant mechanisms because such disruptions have not previously occurred on the scale being considered here. Therefore, the longer-term effects will be more uncertain than the short-term effects. Furthermore, the long-term impact on HIV in particular might be understated because the deaths caused by new infections during the disruption or the development of resistance to treatment during the disruption would not all occur within 5 years. We also do not consider how the increased stress of the health system could continue after the COVID-19 pandemic, when programmes must be reinstituted and demand increases due to new infections acquired during the pandemic. We also do not consider how long-term global changes will affect disease programmes, such as the effect of a global recession, permanent changes to the global medical supply chain, or drug development pipelines. These effects could be profound and dwarf the effects considered here, but it is not currently possible to gauge the full extent of these global changes.

Overall, we argue that although the precise size of the effect on HIV, tuberculosis, and malaria is uncertain, it is reasonable to anticipate the types of disruption and magnitude of impact described here. We have not aimed to produce granular predictions for any particular setting, but instead sought to provide a general outlook of the relative impact of different types of direct and indirect impact of the COVID-19 pandemic and its response, to help establish priorities for maintaining services.

To create a simple analysis that is useful to those in as many different settings as possible, we have considered just two case study country settings for each disease, such that most countries will be on a continuum between these settings for each disease. However, many factors will determine the impact of these disruptions—eg, the overall level of viral suppression for people with HIV, the relative sizes of the public and private sectors for tuberculosis treatment, and the timing of LLIN distribution. The difficulty in constructing the relevant assumptions and drawing on results from different country settings precludes a more detailed comparison.

A strength of this analysis is the coordinated and comparable way in which the disruptions are represented for each of the different diseases. This complements earlier single-disease analyses that have explored various types of disruption without tying them to particular scenarios for the COVID-19 pandemic.^15^, ^16^, ^17^ In addition, data on disease and health system impact are accumulating and will allow these projections to be updated as the pandemic evolves. However, this analysis is limited by the COVID-19, HIV, malaria, and tuberculosis models being separate and therefore interactions between these diseases that could compound the impacts presented here are missed. Because the models do not share a common representation of the health system or patient behaviour, we do not know whether the assumptions about limitations to services for the different diseases correctly gauge the extent of disruption consistently across the diseases.

In conclusion, disruptions to the services for HIV, tuberculosis, and malaria resulting from the COVID-19 pandemic and its response could lead to a substantial number of additional deaths and years of life lost, especially when considering the years of life lost after the pandemic. In the short term, maintaining the most critical services, specifically treatment for HIV and tuberculosis (new and current patients) and provision of both LLINs and prophylactic treatment for malaria, is a priority for reducing the overall impact of the COVID-19 pandemic. A major focus in the longer term is likely to be improving the resilience of the health system to cope with shock events such as pandemics, and the changes necessary could be far-reaching.^18^
