# Correction to *Lancet Glob Health* 2020; 8: e1132–41

**DOI:** 10.1016/S2214-109X(20)30433-2

**Published:** 2020-10-16

*Hogan AB, Jewell BL, Sherrard-Smith E, et al. Potential impact of the COVID-19 pandemic on HIV, tuberculosis, and malaria in low-income and middle-income countries: a modelling study.* Lancet Glob Health *2020;* 8: *e1132–41*—In figure 2 of this Article, the labels for the blue and dark red categories should have been reversed. This correction has been made as of Oct 16, 2020.

