# Supplementary material for: Potential impact of the COVID-19 pandemic on HIV, tuberculosis, and malaria in low-income and middle-income countries: a modelling study
Source: Lancet Glob Health. 2020 Jul 13;8(9):e1132–41. doi: 10.1016/S2214-109X(20)30288-6 (PMC7357988; doi:10.1016/S2214-109X(20)30288-6)
Supplement: Supplementary appendix [file mmc1.pdf]

THE LANCET
Global Health

Supplementary appendix

This appendix formed part of the original submission and has been peer reviewed. We post it as supplied by the authors.

Supplement to: Hogan AB, Jewell BL, Sherrard-Smith E, et al. Potential impact of the COVID-19 pandemic on HIV, tuberculosis, and malaria in low-income and middle-income countries: a modelling study. *Lancet Glob Health* 2020; published online July 13. http://dx.doi.org/10.1016/S2214-109X(20)30288-6.

Supplementary Material

Potential impact of the COVID-19 pandemic on HIV, TB and malaria in low- and middle-income countries

Supplementary results

Table S1: Numbers of additional deaths due and life-years lost per million capita in the year 2020 for the simulation with COVID-19 compared to the simulation without COVID-19. The Country Setting refers to the different settings that are being explored in each model (see Methods).

Covid-19 Pandemic Scenario		Covid-19	HIV		TB		Malaria	
			Country Setting 1	Country Setting 2	Country Setting 1	Country Setting 2	Country Setting 1	Country Setting 2
Additional Deaths	No Action	5,965	161	84	-1	0	1,978	1,018
	Mitigation	4,393	21	11	51	3	2,041	1,225
	Suppression-Lift	5,965	45	23	21	1	2,009	1,092
	Well-Managed suppression	0	11	5	56	3	82	93
	Unmanaged Suppression	0	42	21	69	3	2,149	1,348
Additional Life-Years Lost	No Action	121,069	3,060	1,593	-52	-8	102,253	53,955
	Mitigation	86,348	396	205	1,809	88	105,525	64,906
	Suppression-Lift	121,065	857	445	726	29	103,871	57,873
	Well-Managed suppression	0	207	101	1,979	98	4259	4,947
	Unmanaged Suppression	10	794	402	2,416	111	111,097	71,427

Table S2: Numbers of additional deaths due and life-years lost per million capita in the five-year period 2020–2024 for the simulation with COVID-19 compared to the simulation without COVID-19. The ‘Country Setting’ refers to the different settings that are being explored in each model (see Methods).

Covid-19 Pandemic Scenario		Covid-19	HIV		TB		Malaria	
			**Country Setting 1**	**Country Setting 2**	**Country Setting 1**	**Country Setting 2**	**Country Setting 1**	**Country Setting 2**
**Additional Deaths**	**No Action**	5,965	596	293	29	1	287	464
	**Mitigation**	4,393	160	83	362	11	355	667
	**Suppression-Lift**	5,965	612	306	127	4	323	553
	**Well-Managed Suppression**	0	69	33	784	25	79	132
	**Unmanaged Suppression**	0	421	211	987	31	474	1,018
**Additional Life-Years Lost**	**No Action**	121,069	10,992	5,459	1,041	22	14,658	24,199
	**Mitigation**	86,348	2,954	1,541	12,689	381	18,243	34,890
	**Suppression-Lift**	121,065	11,165	5,639	4,446	122	16,570	28,913
	**Well-Managed Suppression**	0	1,227	596	27,460	866	4,082	6,980
	**Unmanaged Suppression**	10	7,589	3,825	34,573	1,115	24,378	53,360

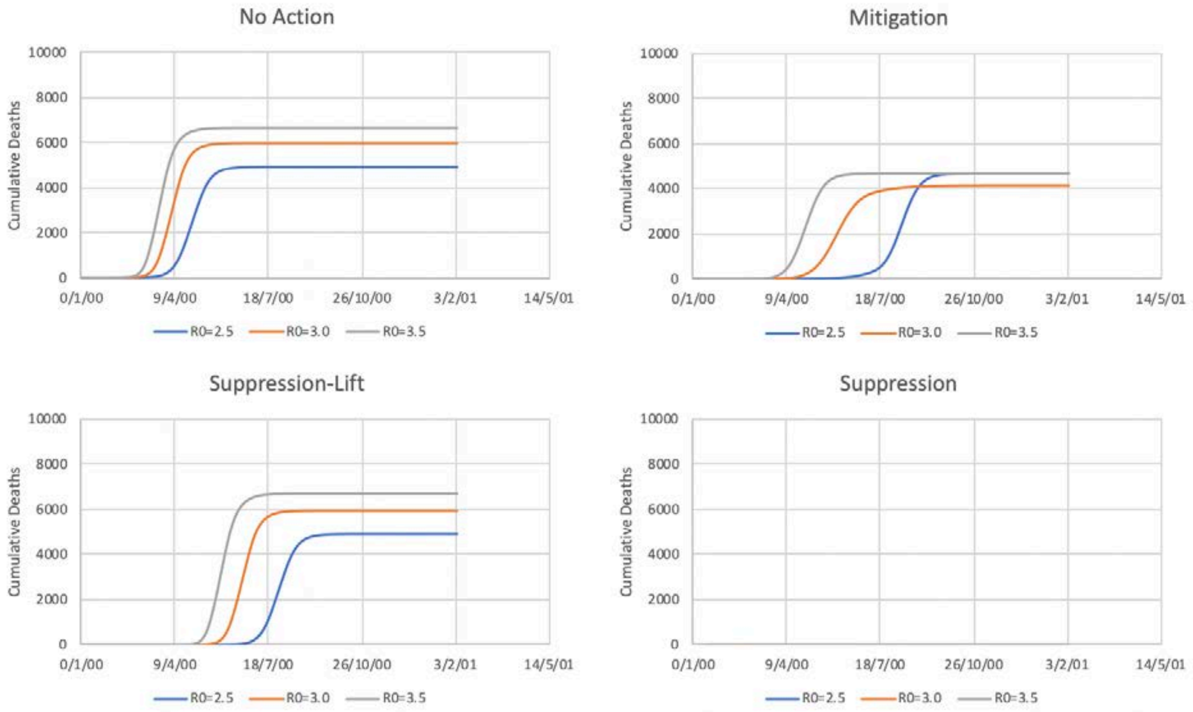

Figure S1: Cumulative deaths due to COVID-19 per million population under the different scenarios and values for R0.

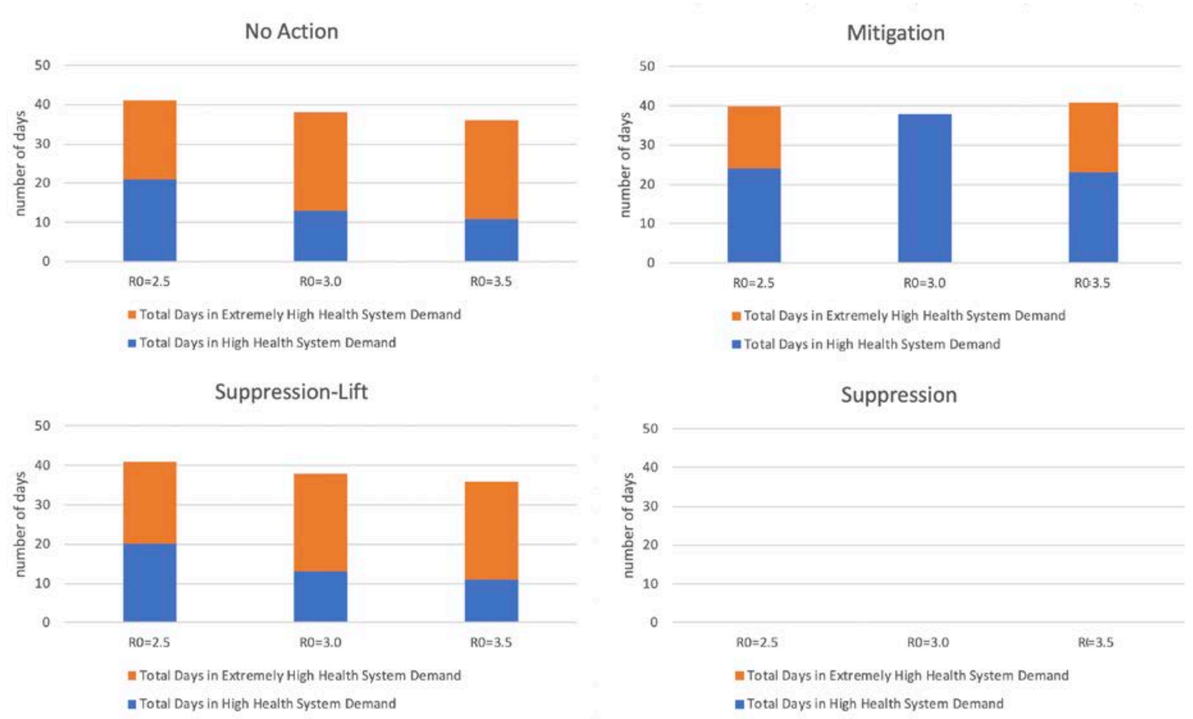

Figure S2: The number of days in the different phase of demand on the health system, according to the value of R_0 for SARS-CoV-2. Note that for the 'No Action' and 'Suppression-Lift' scenarios, the smaller value of R_0 leads to longer period of High or Extremely High Health-system demand. The parameterisation of the effect of the 'Mitigation' scenario was chosen so as a period of 'Extremely High' demand is avoided when R_0=3.0. It would not be expected for the same to be reproduced under different values for R_0.

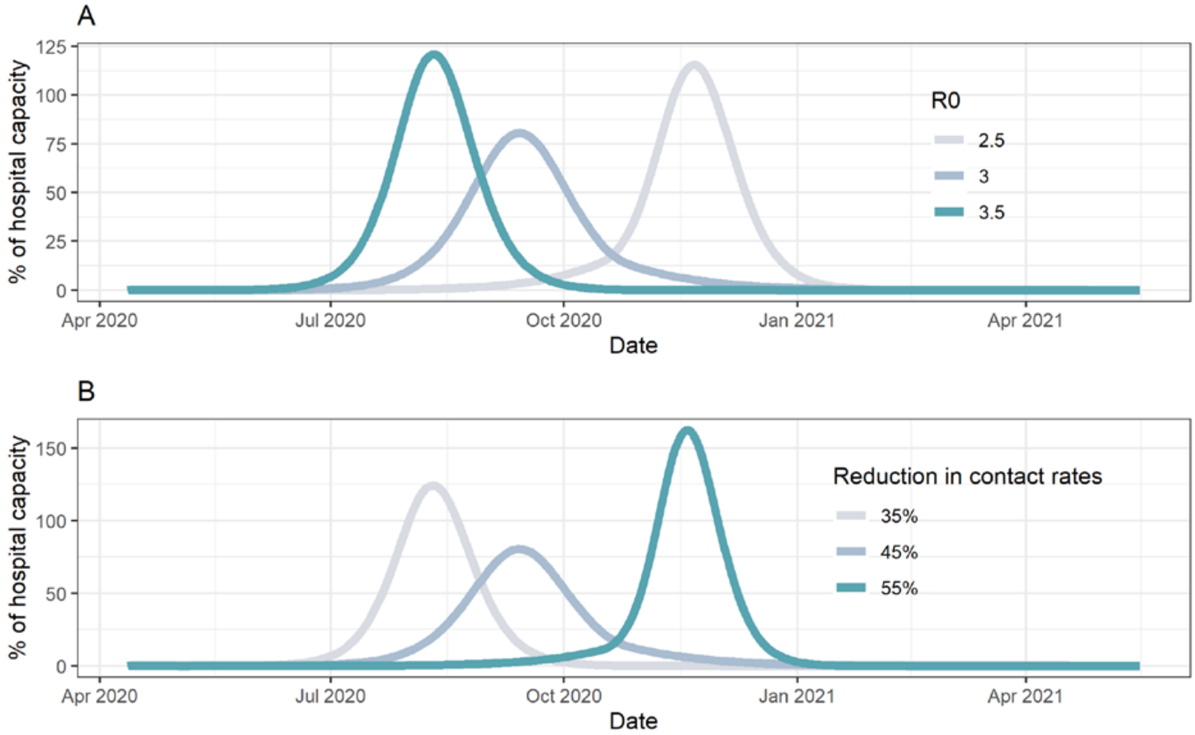

Figure S3: Relative healthcare demand under different assumed R_0s and degrees of mitigation. **A** shows 6 months of mitigation involving a 45% reduction in contact rates for R_0=2.5, 3 (baseline assumption) and 3.5. **B** shows scenarios with R_0=3 and 6 months of mitigation involving a for reduction in contact rates of 35%, 45% (baseline assumption) and 55%.

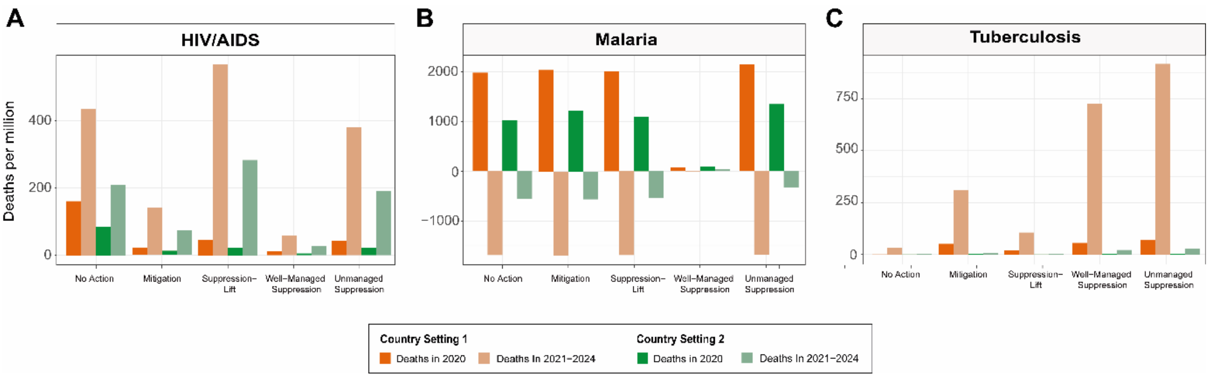

Figure S4: Excess deaths per million population due to HIV, TB and malaria (A, B and C respectively) during 2020 (dark green and dark orange) and over the period 2021–2024 (light green and light orange), under each scenario for the COVID-19 pandemic, and for each of Country Setting 1 and Country Setting 2.

Supplementary methods and discussion

COVID-19 model

To assess the potential impact of different COVID-19 epidemic control scenarios and their associated indirect impacts on the burden of other diseases, we utilised a previously developed age-structured SEIR model of SARS-CoV-2 transmission that explicitly captures consideration of disease severity (whether an individual is asymptomatic, has mild disease, requires general hospital admission or ICU care) and the associated healthcare requirements (high flow oxygen for those in general hospital beds, high flow oxygen and mechanical ventilation for those in ICU beds).[1,2]

The model incorporates age-specific disease severity using age-dependent probabilities for the proportion of infections that result in disease requiring hospitalisation (and hence the need for treatment with high-flow oxygen), the proportion developing severe pneumonia disease and hence requiring intensive care (for whom ~80% require mechanical ventilation) and the risk of mortality. Model parameters for transmission and disease progression are based on analysis of data from China and the UK.[1,2] To produce simulations representative of the LMIC settings considered here, the model was calibrated to typical social contact patterns observed within surveys in sub-Saharan Africa (here we used a survey from Zimbabwe) which show less substantial declines in contact rates by age compared to the UK, and used the demographic schedules of Nigeria.[3] Life-years lost were calculated under this demography using the corresponding life tables.

The model structure is briefly described here (and in Figures S4 and S5). Note that for all states except I_{Mild} described here, two compartments are used to generate a gamma distribution of waiting times. Individuals begin as Susceptible (S) and upon infection, progress to the Exposed (latent infection, E) compartment. From there, individuals either progress to I_{Mild}, which are individuals either asymptomatic or not ill enough to require hospitalisation and medical intervention, or I_{Case}, which represents individuals who are symptomatic and whose disease will eventually become severe enough to require hospitalisation – the proportion of asymptomatic/mild to severe cases varies with age. Individuals in the I_{Mild} compartment are assumed to all recover, upon which they move to the Recovered (R) compartment. Individuals in I_{Case} then progress (in an age-dependent manner) to either $I_{Hospital}$, which represents individuals requiring a general hospital bed and supportive oxygen therapy, or I_{ICU}, representing individuals requiring either a higher degree of oxygen therapy or high flow oxygen and mechanical ventilation. Individuals in both sets of compartments are assumed to die, with the probability of mortality occurring in both a severity (higher for those requiring ICU care than for those requiring a general hospital bed) and age-dependent manner. For those individuals that survive, those in $I_{Hospital}$ then move to the R compartment, whilst those previously in I_{ICU} spend a brief period in a general hospital bed (I_{Rec}) to recover from their stay in the ICU, before moving to the R compartment. Given the short-term dynamics we do not model births, deaths or aging.

In addition, we explicitly model the availability of healthcare within the model (Figure S5). Specifically, at each timepoint, we estimate the number of hospital and ICU beds available given currently levels of occupancy and use that to determine whether individuals newly requiring hospitalisation are able to receive the appropriate care. We assume significant excess mortality associated with not receiving the appropriate care for COVID-19 (detailed in the squire R package documentation) and assume patterns of healthcare seeking behaviour are homogeneous across the entire population. To capture the likely constraints within a health system we contrasted this demand for healthcare predicted by the model under different control scenarios with a representative level of capacity using the median estimated provision of hospital beds and intensive care units for a low-income country.[1,2] This threshold was chosen on the basis that, although many countries in sub-Saharan Africa are lower-middle-income and therefore likely to have a higher total number of hospital beds and intensive care units, access to high pressure oxygen and mechanical ventilation within hospitals is proportionately lower than in high-income settings. During the course of a projected scenario, as healthcare capacity is exceeded, individuals requiring either mechanical ventilation or high-pressure oxygen who are not able to receive these interventions are then subject to a substantially higher degree of mortality, leading to excess mortality during time-periods in which health systems are overwhelmed. Full details, code and parameterisation are available.[2]

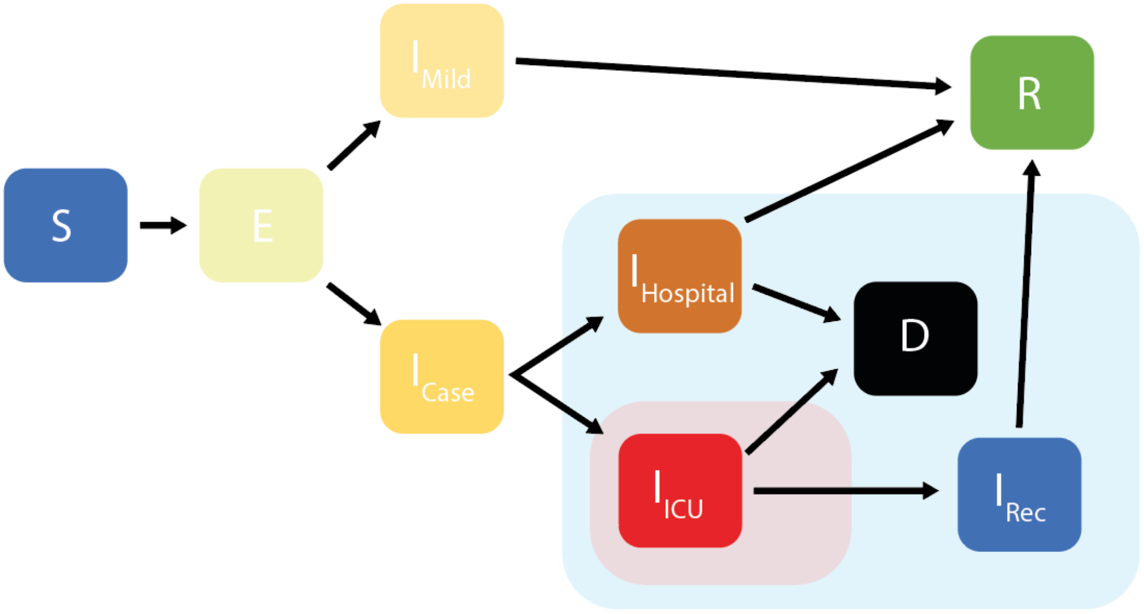

Figure S4: An age-structured SEIR model of SARS-CoV2 transmission that explicitly captures different disease severity and the different associated passages through health systems. In the diagram above, S = Susceptible, E = Exposed (latent infection), I_{Mild} = Mild Infections (those not severe enough to require hospitalisation), I_{Case} = Infections Requiring Hospitalisation (but during the period in which their illness has not yet progressed to being severe enough to require hospitalisation), $I_{Hospital}$ = Hospitalised Infections (requiring a general hospital bed/oxygen support only), I_{ICU} = Hospitalised Infections (requiring an ICU bed and either high flow oxygen or high flow oxygen and mechanical ventilation), I_{Rec} = Hospitalised Infections (requiring a general hospital bed whilst recovering from a stay in the ICU), R = Recoveries and D = Deaths. The boxes indicate compartments associated with health systems, with the blue box indicating compartments related to hospitalisation and occupation of a general hospital bed. The red box indicates compartments related to hospitalisation and that occupy an ICU hospital bed.

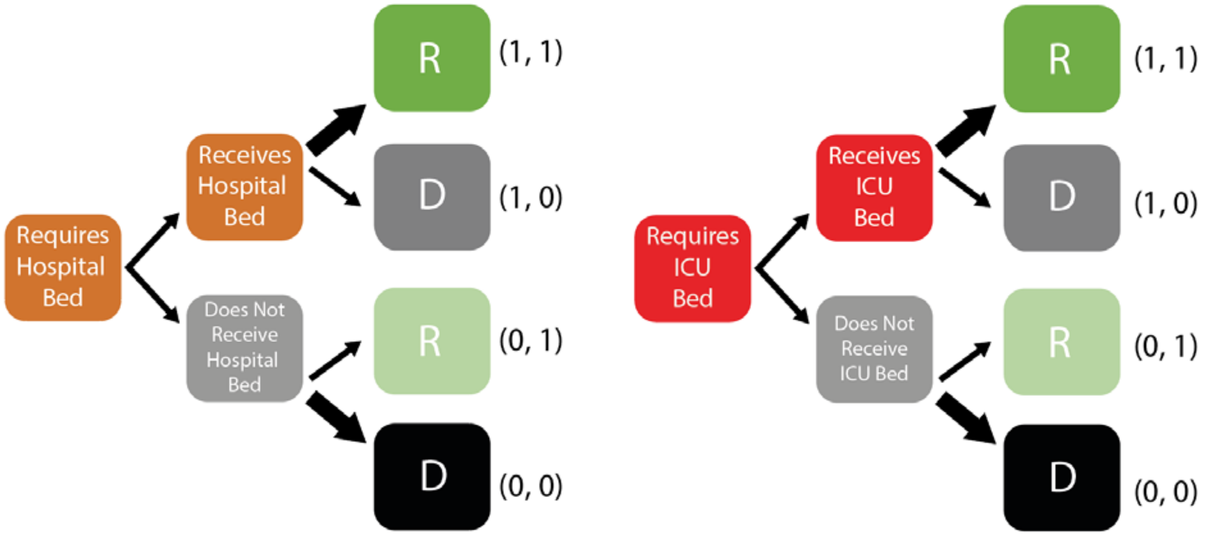

Figure S5: The decision tree cascade utilised to explicitly incorporate considerations of healthcare capacity and associated excess mortality. Within the SEIR modelling framework utilised here, healthcare capacity (expressed as the number of hospital beds and the number of ICU beds) is tracked explicitly. At each timestep, individuals requiring each of these different types of beds are assigned available beds (if any), in an age-independent manner. Those receiving a bed (and hence appropriate care) are subject to a lower probability of mortality those who do not. Notation to the right-hand side describes whether an individual has received appropriate care (0 or 1, first number) and whether that individual subsequently recovers or dies (0 or 1, second number).

Representative scenarios were simulated using a basic reproduction number of 3 representing a 3.5 day doubling time in cases and deaths, which is thought to be reflective of many trajectories currently observed globally.[4] Once a threshold of 0.1 deaths per million (approximately reflecting the COVID-19 level of mortality observed in many countries in Africa to date) is exceeded, the pandemic trajectory follows four potential scenarios:

- 'No Action'. Here no direct action is taken but contact rates are reduced by 20% relative to baseline according to assumed behaviour change in the face of the pandemic even in the absence of specific, coordinated public health interventions.
- 'Mitigation'. Here through combinations of isolation and social distancing contact rates are reduced by 45% for a period of six months after which infections fall to low levels and contact rates return to pre-pandemic levels. This scenario approximates the maximum reduction in the final size of the pandemic that can be achieved whilst generating sufficient levels of immunity capable of preventing a second wave once measures are lifted (assuming infection leads to high levels of immunity from reinfection) and thus produces the lowest final numbers of COVID-19 infections of the three strategies that do not involve indefinite suppression.
- 'Suppression-Lift'. Here the stringent 'lockdown' type interventions implemented by many countries are represented by a reduction in contact rates of 75%. This reduction is maintained for two months at which point it is lifted and contact rates return to 80% of their pre-pandemic levels (i.e. the levels simulated within our 'No Action' scenario) for the remainder of the pandemic.
- 'Suppression'. Here stringent suppression-targeting interventions are implemented to reduce contact rates by 75% and these are maintained indefinitely in the hope that a pharmaceutical intervention (e.g. effective vaccine) can be developed and deployed. We ran this scenario for 12 months but note that at the end of this period, lifting suppression in the absence of such a pharmaceutical intervention would lead to a second wave of equivalent size as in the 'Suppression-Lift' scenario. We assumed both 'Well-Managed Suppression' and 'Unmanaged Suppression' scenarios to have the same effect on COVID-19, but with distinct effects on endemic diseases, as listed in Table 2 in the main text.

HIV model

We used an established deterministic mathematical model of HIV transmission to quantify the impact of disruptions in representative settings, based on original parameterisations for South Africa and Malawi.[5,6] The assumptions made for the disruptions in HIV services were generated through discussion with the HIV Modelling Consortium, although there is no endorsement for any one particular set of assumptions. The disruptions incurred during when there is a 'Mitigation' or 'Well-Managed Suppression' intervention are intended to correspond to what could occur through a combination of the intentional scaling back of services (for voluntary medical male circumcision (VMMC) and pre-exposure prophylaxis (PrEP)), patients on antiretroviral therapy (ART) being less able or willing to gain timely refill prescriptions, and a reduction in time spent away from the home leading to reduced acquisition of new sexual partners. The disruptions incurred during when there is a 'Unmanaged Suppression' intervention are intended to correspond to what could occur when, in addition, persons postpone seeking HIV testing or linking to ART programs or PrEP programs and a small proportion of those on ART do not continue to access medication. The disruptions incurred during when there is a 'High Demand' on the health system correspond to what could happen if viral loading testing is not available (due to the machines having been repurposed) such that a fraction of those of ART become virally unsuppressed, when otherwise detection would have to lead to intervention and re-suppression; and also when the health system is unable to accept new patients to start ART, PrEP or VMMC. During when there is 'Extremely High Demand', it is assumed that disruptions in the supply of ARVs and condoms could lead to a fraction of those on ART not being able to access any medication and for condoms to be used less frequently than otherwise.

In this model, persons with temporarily unsuppressed viral loads are more likely to transmit HIV (2.5 times more, compared to those on ART with no interruption) but do not suffer other adverse consequences. For those that have an interruption in ART, there is both a higher risk of transmission and of dying. Results are most sensitive to the risk of dying for people living with HIV (PLHIV) who have had their ART supply interrupted. This quantity is not well known. We modified the progression to AIDS and death for that group such that the 12-month risk of death would be ~2.91% (similar to the 3.3 / 100 pyar risk of death or opportunistic disease among those experiencing a treatment interruption in a clinical trial)[7] and mean net survival would be ~14 years (approximately the survival time for HIV-positive persons who have never been on ART).[8] We also note that the monthly risk of death is likely to increase over time as individuals accrue time off of ART, however, this is not represented in the model. When the supply of ART is resumed at the beginning of the recovery period, those persons who have not progressed to AIDS re-initiate ART and do not suffer any long-term health consequences

from the interruption. However, those who have progressed to AIDS by that time do not gain any benefit if they are restarted on ART.

The assumption for the 10% reduction in the risk of acquiring HIV is motivated by noting that ‘stay at home’ messages may lead to fewer new sexual partnerships being formed but a somewhat increased risk in transmission among partners who cohabit. It is estimated that 30% of transmission in a given year is between those in long-term partnerships, many of whom will co-habit.[9] So, if transmission in the community is decreased by ~60%, but transmission risk between those in the household is doubled, the net effect is a ~10% decrease in overall transmission.

Other important limitations to note include: (i) no interaction between HIV and COVID-19 infection are incorporated – that is, PLHIV are not assumed to be more or less likely to acquire or die from COVID-19; (ii) the effect of disruption on the risk of mother-to-child transmission is not incorporated; and (iii) possible increases in drug resistance due to ART regimens being disrupted are not incorporated. In each case, this would lead to greater impact of the disruptions in increased adverse outcomes for HIV, particularly over longer timescales. PrEP coverage is so low in the settings modelled that disruption to PrEP has no effect in the model.

TB model
A compartmental, deterministic model of TB transmission dynamics was used to capture the impact of the COVID response on TB.[10] Expert opinion was gathered on the extent of potential disruptions (presented in Cilloni *et al.*).[11]

The model incorporates the acquisition and transmission of rifampicin resistance, as well as the role of HIV in driving TB incidence. It also includes, in a simple way, interactions between symptomatic patients and the health system, capturing (for example) the potential for repeat visits to a care provider before TB is diagnosed, and the ongoing transmission that occurs as a result of this diagnostic delay. The model does not capture the dynamics of HIV or ART coverage, instead taking this as a fixed input, informed by UNAIDS estimates.[12] The model is parameterised using World Health Organization (WHO) estimates for incidence in 2018 for the proportion of TB cases that were HIV-coinfected and for the proportion of incident TB that was rifampicin-resistant.[13] As the model does not have an age-structure, years of life lost are estimated by comparing total years-of-life lived with and without the disruptions being applied.

In terms of effect on interpersonal contact rates, while the ‘Mitigation’ and ‘Suppression’ scenarios will reduce opportunities for community-based transmission, they will also intensify and prolong household exposure to infectious TB. Combining these factors leads to an estimate of a reduction in overall transmission of 10%. The model also captures an initial patient delay before first presenting for care; it is assumed that this delay is extended during the period of mitigation/suppression intervention, to reflect associated difficulties in accessing care. Molecular diagnostic tools such as Xpert MTB/RIF are widely used for TB diagnosis but may be increasingly used for testing for SARS-CoV-2 instead.[14,15] Meanwhile, persons with symptoms are also expected to reduce their care seeking. Accordingly, we assume that the probability of diagnosis drops by 70% under the Mitigation and Suppression scenarios, allowing for some degree of clinical diagnosis in the absence of diagnostic tools. Moreover, Xpert MTB/RIF is used to detect rifampicin resistance, a function that cannot be performed using clinical diagnosis. Therefore, the proportion of cases having a drug sensitivity result is assumed to decline by 50% under high demand on the health system, or to 0% under very high demand. Finally, disruptions in the supplies of TB drugs may be expected when the health system comes under stress.[16] In extreme conditions, drug stock outs may occur, leading to only a proportion of TB cases being able to access treatment. Here it is assumed that only 25% have access to treatment under such conditions. The model does not address potential interactions between COVID-19 and TB, although there is some early evidence for potential risks of increased severity of COVID-19, associated with pre-existing TB infection.[17] The modelling analysis presented here complements parallel analysis being conducted by the Stop TB Partnership that examines these TB dynamics at the level of the care cascade, as well as extending it to the global level.[18]

Malaria model
An individual-based transmission dynamics model of malaria was used to predict the number of malaria deaths and lives saved under the COVID-19 scenario.[19] Models were parameterised using 2017 malaria prevalence and the LLIN usage estimated at the administrative 1-unit level from the Malaria Atlas Project with LLIN usage expected to remain at the same level in the next LLIN mass campaign.[20] The level of insecticide resistance in the local mosquito population (which affects the effectiveness of controls) was estimated for each administrative unit from data collated by the WHO and combined with results from experimental hut trials to predict the

epidemiological impact.[21,22] The number of deaths and life-years lost were estimated from the incidence of severe disease and the proportion of clinical cases receiving treatment.[19]

COVID-19 could increase malaria morbidity and mortality by impeding routine prevention activities whilst also reducing treatment of clinical-cases, worsening health outcomes. The assumptions for the level of disruptions to LLIN distributions were based on information from experts from the WHO and within country health programs, whereas parameters for SMC and treatment were informed by a recent WHO modelling analysis.[23] Early reports suggest mass distribution of LLNs may be delayed as countries increase social distancing to reduce the spread of COVID-19. In many countries these campaigns typically involve large gatherings of people who congregate centrally to receive their LLINs. Similarly, Seasonal Malaria Chemoprevention (SMC) activities, which distribute antimalarial medicine to children door-to-door in areas of highly seasonal transmission to prevent malaria illness, may also be disrupted. It is therefore assumed that these activities will be halted in 2020 when health systems are overburdened ('High' and 'Extremely High Demand') and during any unmanaged COVID-19 suppression period. It may be possible to conduct both LLIN mass distribution and SMC during the Mitigation or Well-Managed Suppression COVID-19 scenarios, although population coverage (those receiving LLINs or SMC treatments) are likely to be reduced (here assumed to be 50% of 2017 levels). LLIN mass campaigns typically occur every three years.

We examined the impact in two settings that were both assumed to have had mass distribution of LLINs planned for 2020 (Country Setting 1 quarter 1; Country Setting 2 in quarters 2–3). Timing of LLIN distribution and seasonality of transmission was set to represent a typical west-African and east-African country for Country Settings 1 and 2, respectively. We assumed that all previous LLINs were distributed in a mass campaign in 2017 although both countries were also assumed to distribute a low percentage of their LLINs continually through routes such as antenatal clinics. Delayed campaigns were assumed to occur a year later than originally planned and again in 2023. All LLINs distributed were assumed to be standard pyrethroid LLINs. SMC takes place annually in a small region of the Country Setting 1 with 70% of children younger than five years of age receiving a full round of treatment in the counterfactual no COVID-19 scenario (and 35% coverage in the Mitigation and Well-Managed Suppression simulations). The treatment of clinical cases with recommended first-line drugs is likely to be impeded when the health system is at capacity as facilities close. Therefore, we assumed that the proportion of those receiving appropriate prompt treatment remains at 2017 levels unless the health system is in a period of 'High Demand', when that proportion is reduced by 50%. We assumed that no clinical cases of malaria are treated when there is 'Extremely High Demand' on the health system or during the Unmanaged Suppression scenario.

References

1 Walker PGT, Whittaker C, Watson O, *et al.* The Global Impact of COVID-19 and Strategies for Mitigation and Suppression. 2020 DOI:10.25561/77735. *[To be updated before publication]*

2 Walker PG, Watson OJ, Whittaker C, Winskill P, Charles G. squire: SEIR transmission model of COVID-19. 2020. https://mrc-ide.github.io/squire/index.html.

3 United Nations. United Nations World Population Prospects. 2019. https://population.un.org/wpp/ (accessed April 18, 2019).

4 Flaxman S, Mishra S, Gandy A, *et al.* Estimating the number of infections and the impact of non-pharmaceutical interventions on COVID-19 in 11 European countries. 2020 DOI:10.25561/77731.

5 Cremin I, Alsallaq R, Dybul M, Piot P, Garnett G, Hallett TB. The new role of antiretrovirals in combination HIV prevention: A mathematical modelling analysis. *AIDS* 2013; **27**: 447–58.

6 Smith JA, Anderson SJ, Harris KL, *et al.* Maximising HIV prevention by balancing the opportunities of today with the promises of tomorrow: a modelling study. *Lancet HIV* 2016; **3**: e289–96.

7 Strategies for Management of Antiretroviral Therapy (SMART) Study Group, El-Sadr WM, Lundgren JD, *et al.* CD4+ count-guided interruption of antiretroviral treatment. *N Engl J Med* 2006; **355**: 2283–96.

8 Todd J, Glynn JR, Marston M, *et al.* Time from HIV seroconversion to death: A collaborative analysis of eight studies in six low and middle-income countries before highly active antiretroviral therapy. *Aids* 2007; **21**: 1–15.

9 Chemaitelly H, Shelton JD, Hallett TB, Abu-Raddad LJ. Only a fraction of new HIV infections occur within identifiable stable discordant couples in sub-Saharan Africa. *AIDS* 2013; **27**: 251–60.

10 Vesga JF, Hallett TB, Reid MJA, *et al.* Assessing tuberculosis control priorities in high-burden settings: a modelling approach. *Lancet Glob Heal* 2019; **7**: e585–95.

11 Cilloni L, Fu H, Vesga JF, *et al.* The potential impact of the COVID-19 pandemic on tuberculosis: a modelling analysis. *medRxiv* 2020; : 1–11. *[To be updated before publication]*
12 UNAIDS. AIDSInfo. https://aidsinfo.unaids.org (accessed April 21, 2020).
13 World Health Organization. Global Tuberculosis Report 2019. 2019. https://www.who.int/tb/publications/global_report/en/ (accessed May 12, 2020).
14 Lawn SD, Nicol MP. Xpert MTB/RIF assay: development, evaluation and implementation of a new rapid molecular diagnostic for tuberculosis and rifampicin resistance. *Future Microbiol* 2011; **6**: 1067–82.
15 World Health Organization Regional Office for Europe. Rapid communication on the role of the GeneXpert ® platform for rapid molecular testing for SARS-CoV-2 in the WHO European Region European Laboratory Initiative on TB, HIV and Viral Hepatitis. 2020 http://www.euro.who.int/en/health-topics/communicable-diseases/tuberculosis/publications/2020/rapid-communication-on-the-role-of-the-genexpert-platform-for-rapid-molecular-testing-for-sars-cov-2-in-the-who-european-region-2020.
16 Arinaminpathy N, Dye C. Health in financial crises: Economic recession and tuberculosis in Central and Eastern Europe. *J R Soc Interface* 2010; **7**: 1559–69.
17 Liu Y, Bi L, Chen Y, *et al.* Active or latent tuberculosis increases susceptibility to COVID-19 and disease severity. *medRxiv* 2020; : 2020.03.10.20033795.
18 Stop TB Partnership. The potential impact of the COVID-19 response on tuberculosis in high-burden countries: a modelling analysis. 2020 http://www.stoptb.org/assets/documents/news/Modeling Report_1 May 2020_FINAL.pdf.
19 Griffin JT, Bhatt S, Sinka ME, *et al.* Potential for reduction of burden and local elimination of malaria by reducing Plasmodium falciparum malaria transmission: A mathematical modelling study. *Lancet Infect Dis* 2016; **16**: 465–72.
20 Malaria Atlas Project. MAP. 2016. https://map.ox.ac.uk/ (accessed April 18, 2019).
21 World Health Organization. Malaria Threats Map. 2020. https://apps.who.int/malaria/maps/threats/ (accessed May 12, 2020).
22 Churcher TS, Lissenden N, Griffin JT, Worrall E, Ranson H. The impact of pyrethroid resistance on the efficacy and effectiveness of bednets for malaria control in Africa. *Elife* 2016; **5**. DOI:10.7554/eLife.16090.
23 World Health Organization Global Malaria Programme. The potential impact of health service disruptions on the burden of malaria: a modelling analysis for countries in sub-Saharan Africa. 2020 www.who.int/publications-detail/the-potential-impact-of-health-service-disruptions-on-the-burden-of-malaria (accessed June 6, 2020).
